# Modulation of time in Parkinson’s disease: a review and perspective on cognitive rehabilitation

**DOI:** 10.3389/fpsyt.2024.1379496

**Published:** 2024-04-15

**Authors:** Motoyasu Honma, Yasuo Terao

**Affiliations:** ^1^Department of Physiology, Showa University School of Medicine, Tokyo, Japan; ^2^Department of Medical Physiology, Kyorin University of School of Medicine, Tokyo, Japan

**Keywords:** time cognition, learning, Parkinson’s disease, compensatory property, overlapping structure, rehabilitation

## Abstract

Time cognition is an essential function of human life, and the impairment affects a variety of behavioral patterns. Neuropsychological approaches have been widely demonstrated that Parkinson’s disease (PD) impairs time cognitive processing. Many researchers believe that time cognitive deficits are due to the basal ganglia, including the striatum or subthalamic nucleus, which is the pathomechanism of PD, and are considered to produce only transient recovery due to medication effects. In this perspective, we focus on a compensatory property of brain function based on the improved time cognition independent of basal ganglia recovery and an overlapping structure on the neural network based on an improved inhibitory system by time cognitive training, in patients with PD. This perspective may lead to restoring multiple functions through single function training.

## Introduction

From the moment of birth, humans experience a constantly changing environment, creating time representations that are refined as they grow (1, 2). Time cognition is needed not only to make time-related judgments but also in all aspects of daily life, including the control of body movements and perception of music (3, 4). Time is a fundamental element of human consciousness, and efficient encoding of the time properties of the environment is necessary to connect with the outside world and generate adaptive behavior. In other words, humans learn time and grow with time.

Time has no corresponding sensory organ unlike light and sound, suggesting that it is perceived, processed, and generated by various neural networks in the brain. Previous studies have shown that many brain regions, including the dorsal striatum consisting of the putamen and caudate nuclei, the frontal lobe, and the subthalamic nucleus, are involved in time cognition (5), indicating the complexity of time cognitive processing. Although there are many approaches to investigate the mechanisms of time cognition, a neuropsychological approach has significantly contributed to the field. For example, Parkinson’s disease (PD) is characterized by motor impairments mainly caused by dopaminergic abnormalities (6), and also presents with various cognitive dysfunctions such as working memory, response inhibition, and task switching (7–9). Impairment of time cognition is particularly pronounced, and studies in PD patients have strongly suggested that the basal ganglia, including the striatum and subthalamic nucleus (STN), plays a central role in time perception processing (10–14). While many studies examine disease-induced impairments in time cognition, there is very little literature on learning of time cognition. The purpose of this review is to focus on the deficits of time cognition in PD and to discuss the recovery of these deficits from the perspective of learning mechanisms. These considerations suggest a mechanism for the elaboration of time cognitive processing and adaptation to time. This may provide an important perspective on cognitive rehabilitation strategies for PD patients.

## Types of time cognition

**Figure 1:**
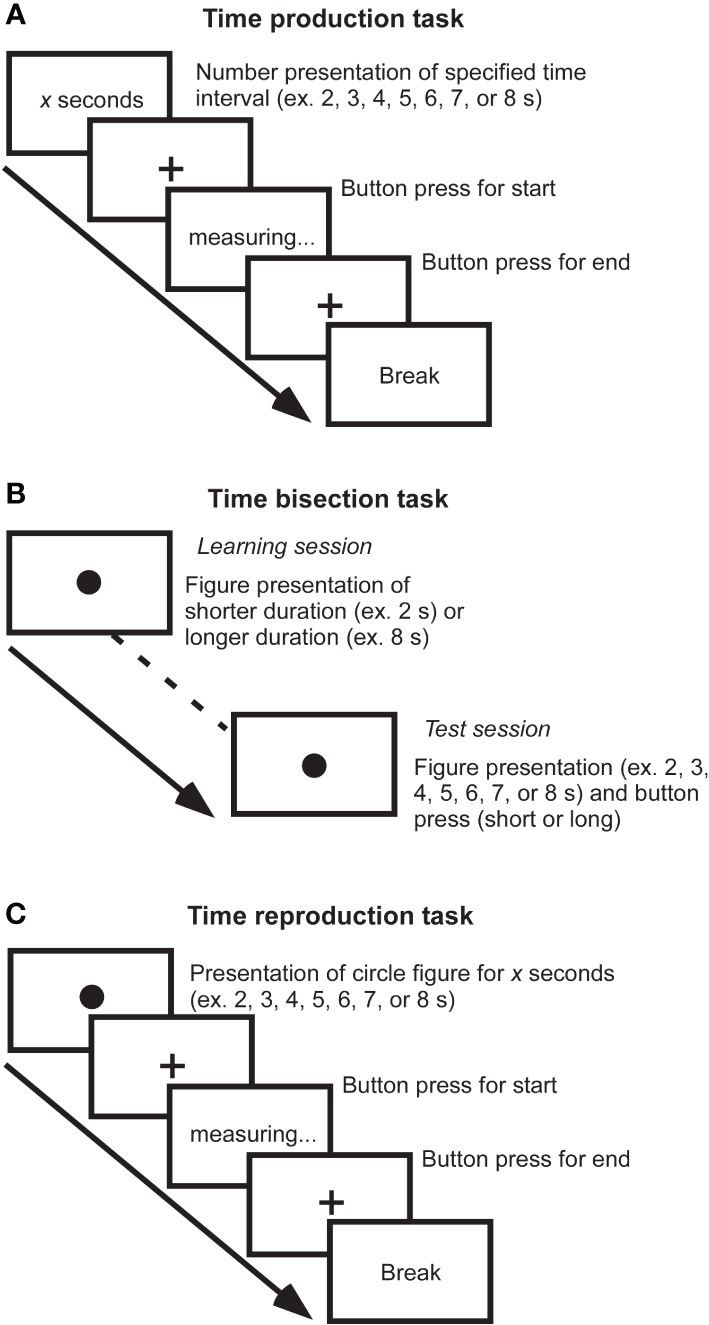
Measurements of time cognition. **(A)** Time production task. The duration to be generated is indicated by a number on the screen, and the participant creates a subjective duration by button pressing the start and end of the measurement. For example, if a participant creates a 6-second duration when a 10-second duration is required, he or she has underestimated the duration. By measuring subjective duration relative to physical duration, it is possible to evaluate an individual’s reference memory created by previous experience. **(B)** Time bisection task. This task is comprised of two sessions: In the learning session, figures with relatively short and relatively long durations are randomly presented to the subjects dozens of times for them to learn the respective durations of figures. Then, in the test session, the participant determines whether the duration of figure presented is closer to which of the two previously learned long/short durations. It requires judging the current duration concerning the immediately preceding learned duration and does not involve much reference memory, allowing the perceptual aspect of the duration to be evaluated. **(C)** Time reproduction task. A figure of specific duration is presented, and the participant reproduces the same duration. For example, even if a participant subjectively judged 6-second duration for physical 10-second duration, if the subject can reproduce the subjective 6-second immediately afterward, the resulting output is the duration of the 10-second. It is possible to assess the working memory of duration rather than reference memory.

Many definitions and models have been proposed to understand time cognition. Time cognition is not a single aspect, and there are at least different types such as disorientation, timing, time order, or time duration. It has been reported that the processing differs depending on types of time cognition (15, 16). Therefore, various studies have focused on different aspects of time cognition. Furthermore, at least the time scale to be studied is divided into three categories: sub-second, close to seconds, and several tens of seconds. It has been suggested that durations of sub-second range are associated with right cerebellar activity, left sensory-motor cortex, and bilateral supplementary motor areas as automatic processing (17), while durations of close to seconds are associated with right dorsolateral prefrontal cortex and right posterior parietal cortex as cognitive processing (18). Thus, there are pieces of evidence for the recruitment of different circuits between short sub-seconds and close to seconds. Furthermore, to investigate aspects of time information processing such as representation, perception, and repetition of time, tasks such as time production, time bisection, and time reproduction tasks are used. Major experimental paradigms are illustrated by a few examples (**Figure 1**). According to the scalar expectancy theory, these tasks can provide insights into the endogenous clock (19).

## Time cognition in Parkinson’s disease

It is known that many patients with neurological and psychiatric disorders have distorted time cognition for duration, which is particularly evident in patients with PD (10–14), autism spectrum disorder (20), schizophrenic disorder (21), and attention-deficit/hyperactivity disorder (22). A possible probable cause is dopamine (**Table 1**). It is thought that a lack of dopamine shortens time estimation, whereas an excess of dopamine tends to prolong time estimation (23). Some studies reported that administering dopamine agonists to PD patients shifted the abnormal time estimation in the normal direction (10). Much of PD patients have striatal deficits involving proteins such as the presynaptic dopamine transporter (DaT), which is responsible for the uptake and transmission of dopamine (24). One study found an association between the underestimation of time duration and DaT levels in the striatum (14).

**Table 1:** List of diseases affecting time duration cognition combined with dopamine.

Articles	Diseases	Dopamine	Time duration cognition
Pastor et al. (10)	Parkinson’s Disease	Deficiency	Shortening
Torta et al., (11)	Parkinson’s Disease	Deficiency	Shortening
Perbal et al. (12)	Parkinson’s Disease	Deficiency	Shortening
Smith et al. (13)	Parkinson’s Disease	Deficiency	Shortening
Honma et al. (14)	Parkinson’s Disease	Deficiency	Shortening
Honma et al. (20)	Autism Spectrum Disorder	Deficiency	Shortening
Mavrogiorgou et al. (21)	Schizophrenic Disorder	Excess	Extension
West et al. (22)	Attention-Deficit/Hyperactivity Disorder	Excess	Extension

Recently, therapy of deep brain stimulation (DBS), which involves continuous electrical stimulation of a target region, has come to be used widely for reducing PD patients’ motor symptoms (25). DBS also affects cognitive functions by altering the function of the basal ganglia-thalamo-cortical loop (26). A study measured time estimation during the on/off states of DBS to the STN in PD patients (27). The results showed no difference between the on/off states in performances of the time bisection and reproduction tasks, although the off state in the production task showed time underestimation. The effect of STN-DBS in the production task suggests that the STN affects reference memory processing (long-term memory). In contrast, the lack of STN-DBS effect on the bisection task suggests that STN does not affect perceptual processing. The effect of STN-DBS was also little in the reproduction task, suggesting that STN-DBS does not affect short-term memory processing. These findings suggest that the STN, as well as the striatum, plays a central role in the processing of time estimation.

## Learning of time cognition

Training with or exposure to specific perceptual stimuli improves task performance and sensitivity to perceptual stimuli. Such changes, called perceptual learning, have been observed to occur in various sensory modalities, including vision, hearing, smell, taste, and touch (28–32). Furthermore, it has been reported that training effects are maintained for months to years (28). Changes due to repeated stimulation may be based on long-term and sustained neural plasticity.

Similar to sensory modalities, learning on time cognition has also been examined. One study reported that time duration can be altered by transcranial magnetic stimulation in healthy participants (33). In the first experiment of the study, participants were asked to produce a subjective 10-second time duration (subjective baseline) in the first test session. In the next false feedback session, they learned the duration of the figure presentation which was 2 sec longer than the subjective baseline (For example, if the subjective baseline was 9.5 seconds, the time duration of a figure presented in the false feedback session would be 11.5 seconds). Before starting the false feedback session, the experimenter told the participant that the duration of the figure to be presented would be exactly 10 seconds. Figures of this newly learned duration were presented 20 times. After the false feedback session, the participant was asked to produce the subjective 10 seconds without cue at every 1 hour. The duration learned in the false feedback session returned to baseline within two hours. This result indicates that even if humans learn an incorrect duration, they naturally return to the originally memorized duration.

In the next experiment, before the false feedback session, persistent neural plasticity was promoted by quadripulse transcranial magnetic stimulation (QPS) over the right dorsolateral prefrontal cortex (DLPFC), temporoparietal junction, or primary motor cortex for 20 minutes. The same false feedback session as in the first experiment was then conducted, and examined how the false duration changed over time. In the results, in the condition where QPS was performed to the DLPFC, but not to the temporoparietal junction or primary motor cortex, the learned false duration was maintained for four hours. This suggests that the new duration is easily consolidated by enhancing the plasticity of the right DLPFC before the false feedback is conducted. Furthermore, the effect persisted for at least one week, implying that a conversion from short-term memory to long-term memory had occurred.

Memory is considered to be stored initially as recent memory within the hippocampus-neocortical network, and as time passes, it is slowly consolidated as remote memory within the neocortex for long-term storage (34, 35). Because it is known that the right DLPFC is implicated in duration production/estimation (36, 37), the maintenance of error time duration over several hours observed by the above study may suggest a memory consolidation of time duration (33). It is possible that memory consolidation, which is inherently transferrable over a longer period, was promoted by enhancing cortical plasticity by QPS. If the right DLPFC is an important node in a conversion process from short-term to long-term memory, QPS over the DLPFC may directly mediate the consolidation process of remote memory for the time duration to be produced.

## Learning of time cognition in Parkinson’s disease

If patients with distorted time estimation learn an accurate time duration, can the time duration be retained? In a study, PD patients learned accurate time duration or spatial length (5 trials) and they were measured the subjective duration and length after a certain time (38). The results showed that PD patients were able to maintain the accurate spatial length, although the duration returned to the original duration (baseline) within 5 minutes. This finding indicates the robustness of the time distortion in PD patients that they are more difficult to modify the time representation, whereas they can flexibly modify spatial representations, suggesting that the learning of time and spatial representations is supported by different neural mechanisms. In addition, another study reported that PD patients undergo training to learn the exact time duration for one month (39). The results showed that the distorted time estimation of PD patients approached normal after the training intervention. The finding suggests that long-term learning can correct the distorted time estimation. In the field of physical rehabilitation, it has been confirmed that the undamaged regions compensate for the motor function of the damaged primary motor cortex (40). Since the Honma et al. (2021) study did not manipulate dopamine or other medications, likely, the basal ganglia, including the striatum and STN, of PD patients have not recovered. This may have allowed other brain regions to compensate for time cognitive functions and approach normal time cognition.

**Figure 2:**
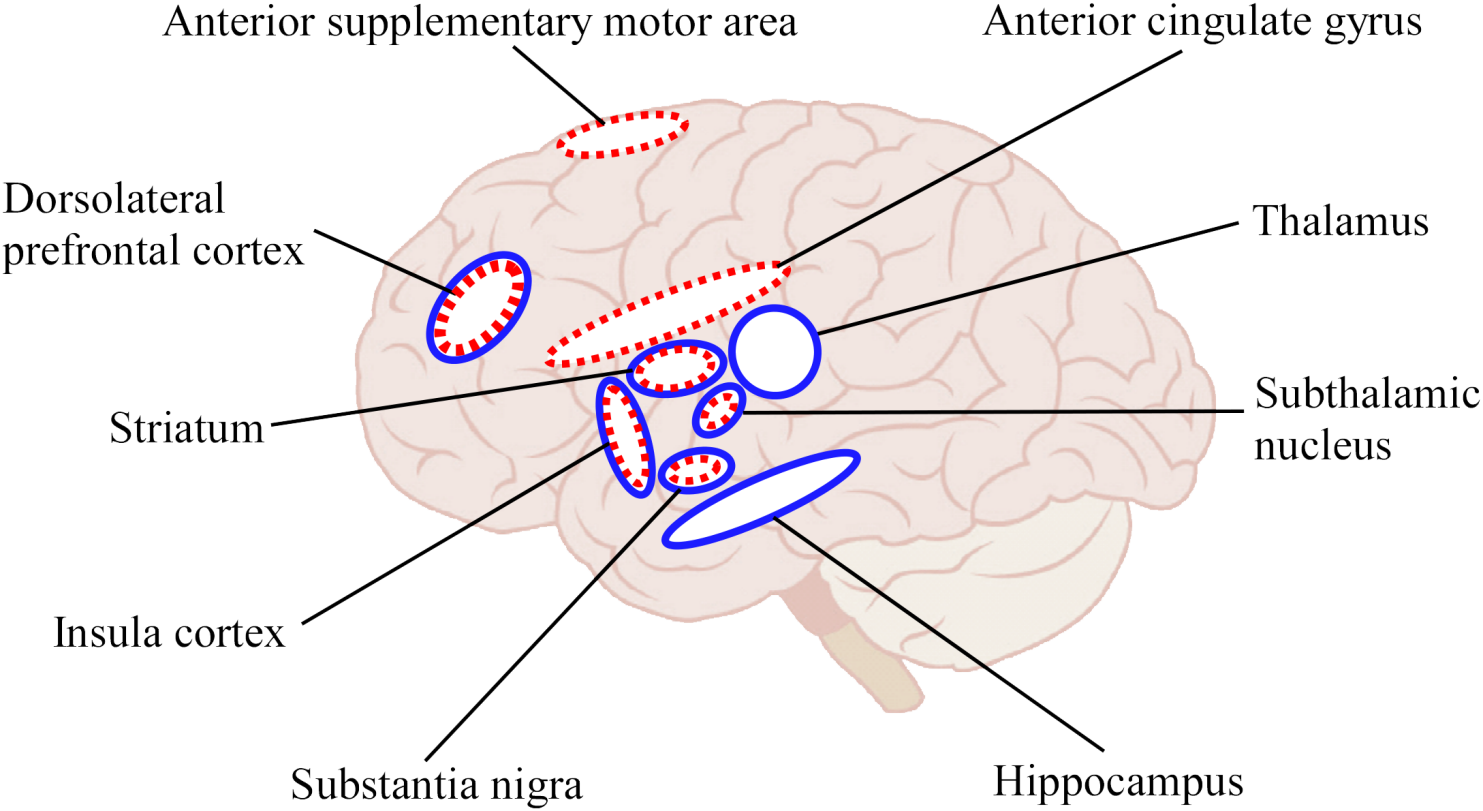
Schematic illustration for brain regions involved in time cognition and response inhibition functions. The blue solid circles indicated the regions related to time cognition function and red dotted circles indicated the regions related to response inhibition function. Of these, the DLPFC, STN, insula cortex, substantia nigra, and striatum overlap in both functions.

The above study (39) also showed concurrent recovery of cognitive functions other than time cognition such as Stroop (41), go/nogo tasks (42), and impulsive disorder tendency (43) by time duration training. In other words, other cognitive functions and mental health improved even though no other than time duration training was performed. More interestingly, the study also showed that performance in N-back task (44) for working memory, tendencies of anxiety (45), and depression (46) were not affected by the time duration training. Possibly the inhibitory system component common to the Stroop, go/nogo tasks, and impulse disorder tendency only was affected. These results suggest that time duration training affected other functions besides time cognition, via brain networks related to time cognition, implying an overlapping structure in the brain related to the inhibitory system. The inhibitory system is mainly involved in the DLPFC, STN, anterior supplementary motor area, insula cortex, anterior cingulate gyrus, substantia nigra, and striatum (47). Among these, DLPFC, STN, insula, striatum, and substantia nigra are common to the time cognitive network (**Figure 2**). This overlapping structure of brain function may be the cause of the improvement in inhibitory system function by time cognitive training. Furthermore, because the STN, substantia nigra, and striatum are impaired by PD, we believe that the DLPFC and/or insula cortex are leading candidate regions that serve as substitutes for the basal ganglia in time cognitive function.

## Future directions

One of the issues for future research is to scrutinize which brain regions are involved in time cognitive learning and to what extent. It is important to identify which regions take the place of the basal ganglia in time cognitive processing, based on the findings noting the compensatory property of the brain function (39). A possible candidate is the DLPFC because the region plays a role in not only estimation but also memory consolidation of time (33, 36). For example, by using functional MRI to measure brain activity before and after time cognitive training, it may be possible to examine which brain regions are responsible for the function of compensatory property.

Although this review mainly focused on PD, it is necessary to consider whether the learning dysfunction of time cognition occurs in other diseases with striatal deficits, such as multiple system atrophy, Huntington’s disease, and progressive supranuclear palsy, etc (48–50). Similar to the above-mentioned research (38, 39), knowing whether time-specific learning dysfunction is present in other diseases would help to elaborate the role of the basal ganglia system in time cognitive learning.

Finally, there is a need to longitudinally investigate at what stage the onset of time cognitive dysfunction and learning disability occurs. Currently, many researchers are focusing on decreased olfactory ability and sleep disorder as precursor symptoms of PD (51–53). Longitudinal studies of the onset timing of disorder might suggest that time cognition is a novel biomarker for predicting PD onset. Furthermore, the degree of time cognitive learning may also vary depending on the stage of progression of the main disease, and then it may be an important perspective in terms of early detection of disease and effectiveness of rehabilitation.

## Conclusion

We outlined the literature showing that the ability to process time cognition and the learning are impaired in patients with PD. The neuropsychological approach suggests that the impairment is mediated by a network centered on the basal ganglia, including the striatum and STN. However, time cognition can be improved by long-term training. In addition, time cognitive training improved not only time cognition itself but also inhibitory functions, despite the absence of any training related to inhibitory functions at all. By viewing from the learning, we pointed out compensatory property due to overlapping structure on the time cognitive function. This perspective may lead to restoring multiple functions through single-function training. When considering cognitive rehabilitation strategies, discovering various types of compensatory properties based on overlapping structures in the brain will provide a new approach to cognitive rehabilitation.

## Author contributions

MH: Conceptualization, Funding acquisition, Validation, Visualization, Writing – original draft, Writing – review & editing. YT: Supervision, Writing – review & editing.
